# Persistence of SARS-CoV-2-Specific Antibodies for 13 Months after Infection

**DOI:** 10.3390/v13112313

**Published:** 2021-11-19

**Authors:** Indrė Kučinskaitė-Kodzė, Martynas Simanavičius, Aistis Šimaitis, Aurelija Žvirblienė

**Affiliations:** 1 Life Sciences Center, Vilnius University, Saulėtekio 7, LT-10257 Vilnius, Lithuania; martynas.simanavicius@bti.vu.lt (M.S.); aurelija.zvirbliene@bti.vu.lt (A.Ž.); 2 The Lithuanian Department of Statistics, Gedimino pr. 29, LT-01104 Vilnius, Lithuania; Aistis.Smaitis@stat.gov.lt

**Keywords:** SARS-CoV-2, serological monitoring, IgG/IgM dynamics, longitudinal study

## Abstract

Background: Dynamics of antibody responses were investigated after a SARS-CoV-2 outbreak in a private company during the first wave of the pandemic. Methods: Workers of a sewing company (Lithuania) with known SARS-CoV-2 RT-PCR result during the outbreak (April 2020) were invited to participate in the study. Virus-specific IgG and IgM were monitored 2, 6 and 13 months after the outbreak via rapid IgG/IgM serological test and SARS-CoV-2 S protein-specific IgG ELISA. Results: Six months after the outbreak, 95% (CI 86–99%) of 59 previously infected individuals had virus-specific antibodies irrespective of the severity of infection. One-third of seropositive individuals had virus-specific IgM along with IgG indicating that IgM may persist for 6 months. Serological testing 13 months after the outbreak included 47 recovered individuals that remained non-vaccinated despite a wide accessibility of COVID-19 vaccines. The seropositivity rate was 83% (CI 69–91%) excluding one case of confirmed asymptomatic reinfection in this group. Between months 6 and 13, IgG levels either declined or remained stable in 31 individual and increased in 7 individuals possibly indicating an exposure to SARS-CoV-2 during the second wave of the pandemic. Conclusions: Detectable levels of SARS-CoV-2-specific antibodies persist up to 13 months after infection for the majority of the cases.

## 1. Introduction

The persistence of antibody levels and duration of a protective immune response after a severe acute respiratory syndrome coronavirus 2 (SARS-CoV-2) infection have not yet been fully studied. In contrast, the dynamics of the humoral immune response during an acute phase of SARS-CoV-2 infection are well-understood [1,2,3]. The seroconversion takes place 6–14 days after the diagnosis of SARS-CoV-2 infection [3,4,5,6]. A significant increase in virus-specific antibody levels is observed at days 16–35 after the onset of symptoms [4,7,8,9,10]. Some studies have reported that antibody levels peak within the first few months, then wane and remain stable for several months, indicating that the immunity may last longer [4,8,10,11,12,13,14,15,16]. Other studies suggest that the levels of SARS-CoV-2-specific IgG are durable and decline after 6–8 months while the number of memory B cells increases within 8 months after infection [17,18]. In line with these observations, cohort studies of healthcare workers from 17 Belgian hospitals indicated the persistence of SARS-CoV-2 S1-specific neutralizing IgG for at least 6 months [19]. Most studies on the persistence of SARS-CoV-2-specific antibodies have been focused to healthcare workers representing a high-risk group in terms of SARS-CoV-2 exposure. A prospective longitudinal study in Spain demonstrated that a significant proportion of healthcare workers previously infected with SARS-CoV-2 maintained a declining seropositivity against both spike (S) and nucleocapsid (N) proteins of SARS-CoV-2 over a period of 9 months: the observed antibody titers decreased in 82% of individuals and remained stable in 13.1% of individuals [16]. The cohort study conducted in the UK demonstrated that the levels of antibodies against the SARS-CoV-2 N protein declined by 31.3% over a period of 3 months [20]. In contrast, another cohort study of healthcare workers in the UK showed that the seropositivity against the SARS-CoV-2 S protein remained stable in 94% (95% credibility interval [CrI] 91–96%) of participants for 6 months [14]. The most recent studies show detectable antibody responses to the SARS-CoV-2 S protein for 12–13 months [13,21].

Thus, previous reports suggest the persistence of SARS-CoV-2-specific antibodies up to 13 months after infection. However, due to an increasing accessibility of COVID-19 vaccines and the start of global vaccination it becomes problematic to monitor the persistence of a virus-specific humoral response after a natural infection, since a significant part of the population is covered by vaccination. In addition, very few, if any, longitudinal cohort studies on the SARS-CoV-2 seropositivity have been performed after well-documented outbreaks in private factories or companies where the risk of virus spread is lower and the possibilities for periodic monitoring of the humoral immune response are more challenging as compared to hospitals. A serosurvey of workers recruited after the first wave of the COVID-19 pandemic from 16 sectors and 32 occupations showed that seropositivity rates varied widely across sectors and occupations, reflecting a higher exposure in nursing home and healthcare sectors [22].

In the current study, the persistence of SARS-CoV-2-specific antibodies was investigated in a cohort of workers of a small sewing company located near Vilnius (Lithuania) where an outbreak of SARS-CoV-2 infection was recorded in April 2020. Serological testing at 2 months, 6 months and 13 months after the outbreak revealed a high seropositivity rate and sustainable levels of virus-specific IgG in most study participants with previously confirmed SARS-CoV-2 infection. To the best of our knowledge, this is the first longitudinal serological study in a private company where an outbreak of SARS-CoV-2 infection was recorded during the first wave of the pandemic.

## 2. Materials and Methods

### 2.1. Study Cohort and Sample Collection

The study cohort consisted of 100 workers of the sewing company located in a small town near Vilnius (Lithuania), where an outbreak of SARS-CoV-2 infection was recorded in April 2020. Capillary blood samples were collected from 50 individuals on 17 June 2020 (2 months after the outbreak), from 100 individuals on 20 October 2020 (6 months after the outbreak) and from 97 individuals on 12 May 2021 (13 months after the outbreak). In the first setting (n = 50), the median age of the participants was 46 years (IQR 40–53, range 18–65) and 89.66% of them were female. In the second setting (n = 100), the median age was 46 years (IQR 40–53, range 18–65) and 88.89% of them were female. In the third setting (n = 97), the median age was 47 years (IQR 41–54, range 18–66) and 83.5% of them were female. The basic characteristics of the study cohort are presented in Table 1.

Prior to the study, approval (No. 2020/5-N2˗1231˗710) of the Regional Bioethics committee (Vilnius, Lithuania) was obtained. All participants of the study signed an informed consent form.

### 2.2. Statistical Analysis

Confidence intervals (CI) presented in this article are 95% Agresti‒Coull intervals for binomial distribution with assumption of sample independence. Interval bounds are rounded to the nearest percent.

### 2.3. Laboratory Testing of Blood Specimens

For detection of SAS-CoV-2-specific antibodies in blood specimens, two types of serological tests were used: a rapid serological test for IgG/IgM antibodies targeting the S1 (spike subunit 1) and N (nucleocapsid) proteins of SARS-CoV-2 (AMP Rapid Test SARS-CoV-2, AMP Diagnostics, Ameda Labordiagnostik GmbH, Austria) and a quantitative ELISA for IgG antibodies targeting the S protein “SARS-CoV-2 S IgG QUANT B ELISA” (UAB Imunodiagnostika, Lithuania) approved by the State Health Care Accreditation Agency under the Ministry of Health of the Republic of Lithuania for IVD use. Prior to use in the cohort study, both serological tests were evaluated using well-characterized blood specimens. For this purpose, a total of 150 SARS-CoV-2-negative blood plasma specimens collected from blood donors before April 2019, and a total of 50 blood plasma specimens collected from patients with previously confirmed COVID-19 diagnosis obtained from the Biobank of Vilnius University Hospital Santaros Clinics were used. To evaluate the performance of the selected serological tests, the same specimens were tested in parallel by the FDA-approved ELISA for the detection of IgG to SARS-CoV-2 S1 protein—Anti-SARS-CoV-2 (IgG), EUROIMMUN (Germany). As a reference test, SARS-CoV-2-specific reverse transcription polymerase chain reaction (RT-PCR) was used. The diagnostic sensitivity and specificity of the AMP rapid serological test for specimens collected >10 days after a positive RT-PCR test or the onset of symptoms were 92% (CI 81–97%) and 99.33% (CI 96–100%), respectively. The sensitivity and specificity of “SARS-CoV-2 S IgG QUANT B ELISA” for the same specimens were 96% (CI 86–99%) and 99.33% (CI 96–100%), respectively, while the sensitivity and specificity of the of EUROIMMUN ELISA were 92% (CI 81–97%) and 98.67% (CI 95–99%), respectively. Thus, the performance characteristics of the AMP rapid serological test and the “SARS-CoV-2 S IgG QUANT B ELISA” were comparable to that of EUROIMMUN ELISA. Blood specimens collected within the study were tested in accordance with the manufacturer’s recommendations. For AMP rapid serological tests, undiluted specimens of the capillary blood were used. For “SARS-CoV-2 S IgG QUANT B ELISA”, capillary blood specimens were collected into Microvette^®^ 500 Serum Gel vials (Sarstedt, Germany) to separate blood serum. For the assay, serum specimens were diluted 1:100. The results of “SARS-CoV-2 S IgG QUANT B ELISA” were standardized according to the First WHO International Standard for anti-SARS-CoV-2 immunoglobulin (human) (NIBSC code: 20/136) [23]. The ELISA test result in relative units (RU)/mL is equal to the anti-SARS-CoV-2 immunoglobulin concentration in binding antibody units (BAU)/mL of the WHO International Standard.

## 3. Results

### 3.1. Epidemiological Context

**Figure 1:**
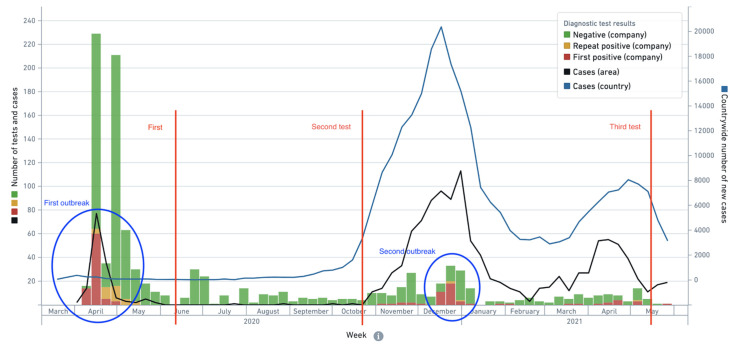
Epidemiological context in the country/local area and the timing of outbreaks of SARS-CoV-2 infection and serological tests in the company. There were two major outbreaks in the company (blue circles), one in April 2020 (two months preceding the first serological test) and another in December 2020 (between the second and third serological tests). There were no cases recorded in the company between the first and the second serological test. Source: VDV IS, the Lithuanian Statistics Department.

The study was performed in a private sewing company in a small town near Vilnius (Lithuania) where the first outbreak of SARS-CoV-2 infection was documented in April 2020. Figure 1 shows the epidemiological context in which serological testing had been conducted. There were two major outbreaks in the company—the first one in April 2020 (when infection cases in the company constituted the majority of cases in the local area) and the second one in December 2020, during the peak of the second wave of the pandemic in the country. In between the first and second serological tests there were no infection cases reported in the company and only a handful in the local area, so it is safe to assume that at time of the second serological test, the participants did not have additional exposure to the virus, whereas at the time of the third test it is highly likely that study participants had exposure to the virus during the second outbreak in between the second and third serological tests.

### 3.2. Characterization of the Study Cohort

The participants of this study were workers of the sewing company that was documented as an outbreak zone of SARS-CoV-2 infection during the first wave of the pandemic. The first case of a symptomatic SARS-CoV-2 infection confirmed using RT-PCR was recorded on 7 April 2020, and the second case on 9 April 2020, followed by a significant increase of infection cases on 13–15 April 2020. In total, 298 out of 300 employees of the company were periodically tested for SARS-CoV-2 infection via RT-PCR within the period of 7–21 April 2020. For 94 of them, the diagnosis of SARS-CoV-2 infection was confirmed by at least one positive RT-PCR test. In addition, 40 workers were tested for SARS-CoV-2-specific IgG/IgM using an AMP rapid serological test on 7–9 April 2020, and 3 of them were found to be seropositive (but RT-PCR negative), possibly indicating the primary source of the infection. Thus, the total number of SARS-CoV-2-positive individuals as determined either using a RT-PCR or serological test was 97. After the outbreak, the workers of the company were invited to participate in our study for monitoring the presence of SARS-CoV-2-specific antibodies. From 17 June 2020 until 12 May 2021, three procedures of serological testing were performed. The first serological testing was performed 2 months after the outbreak (17 June 2020) using a rapid serological test for IgG/IgM antibodies targeting the S1 and N proteins of SARS-CoV-2 (AMP Rapid Test SARS-CoV-2). Fifty workers agreed to participate in the first serological testing, 45 of them with previously diagnosed with SARS-CoV-2 infection. The second testing procedure was performed 6 months after the outbreak (20 October 2020) using the same AMP rapid serological test for SARS-CoV-2-specific IgG/IgM antibodies and a quantitative ELISA for IgG antibodies targeting the S protein (“SARS-CoV-2 S IgG QUANT B ELISA”). The second serological testing included 100 participants, 59 of whom were previously diagnosed with SARS-CoV-2 infection via RT-PCR. The third testing procedure was performed 13 months after the outbreak (12 May 2021) using both tests, the rapid serological test for IgG/IgM and the quantitative ELISA for S-specific IgG. The third serological testing included 97 participants, 63 of whom had tested positive for SARS-CoV-2 using RT-PCR during the outbreak in April 2020. As COVID-19 vaccines were already available before May 2021, 16 participants out of 63 with previously confirmed SARS-CoV-2 infection had been vaccinated with Comirnaty vaccine at the time of the third testing. Thus, the third testing included 47 participants that had recovered from SARS-CoV-2 infection in April 2020 and had not been vaccinated.

### 3.3. Seropositivity Pattern 2 Months after the Outbreak

The first procedure of serological testing was performed 2 months after the outbreak and included 50 participants (median age—46 years, female—89.66%) that had been tested for SARS-CoV-2 infection using RT-PCR in April 2020. For 45 of them, SARS-CoV-2 infection was confirmed by at least one RT-PCR test at the time of the outbreak (Table 2).

**Figure 2:**
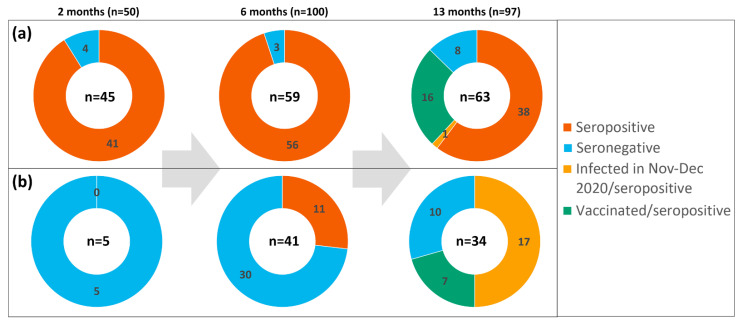
Seropositivity pattern in the groups of study participants tested as SARS-CoV-2-specific RT-PCR positive (**a**) and RT-PCR negative (**b**) during the outbreak in April 2020. On the top of each section, time after the outbreak and the number of study participants subjected to serologic testing are indicated.

Thirty-three of them (73%, CI 58–84%) reported typical symptoms of different severity, from very mild to severe requiring hospitalization and oxygen supply. Twelve participants out of 45 (27%, CI 16–41%) did not feel any symptoms and were categorized as asymptomatic. Five participants of the first serological testing procedure had a negative RT-PCR test and did not report any typical symptoms; they were categorized as SARS-CoV-2-negative. Testing of blood specimens using a rapid serological test revealed 41 seropositive participants among 45 with a confirmed previous SARS-CoV-2 infection: 11 out of 11 with moderate and severe symptoms, 8 out of 9 with mild symptoms, 13 out of 13 with very mild symptoms and 9 out of 12 with asymptomatic SARS-CoV-2 infection. Twenty-two out of 41 seropositive individuals (53%, CI 37–68%) had both IgG and IgM virus-specific antibodies while 19 had only IgG (46%, CI 32–62%). As expected, 5 SARS-CoV-2-negative participants were negative for virus-specific antibodies. Overall, antibodies were found in 91.1% (CI 79–97%) of previously RT-PCR positive individuals (41 out of 45; Figure 2a), with 3 out of 4 who did not have antibodies being in the asymptomatic group.

### 3.4. Seropositivity Pattern 6 Months after the Outbreak

The second procedure of serological testing was performed 6 months after the outbreak and included 100 participants (median age—46 years, female—88.89%) that had been tested for SARS-CoV-2 infection using RT-PCR in April 2020. For 59 of them, previous SARS-CoV-2 infection was confirmed by a positive RT-PCR test in April 2020 (Table 3).

In addition, 3 participants were found to be seropositive but RT-PCR negative in April 2020 when both the RT-PCR and the serological test were applied for some workers of the company. They self-reported very mild symptoms at the time of the outbreak. Testing of blood specimens via a rapid serological test and a quantitative ELISA revealed 56 seropositive individuals among those with previous SARS-CoV-2 infection confirmed using RT-PCR (Figure 2a). Among them, 11 were asymptomatic, 33 had mild or very mild symptoms and 12 had moderate or severe symptoms. From 3 participants who were RT-PCR-negative but seropositive during the outbreak (7–9 April 2020), 2 remained seropositive on 20 October 2020. In addition, 11 seropositive individuals were identified among those who had never had a positive RT-PCR test (Table 3, Figure 2b). Their seropositivity status during the outbreak remains unknown, as only 40 workers out of 300 were tested via serological tests in April 2020. Four out of these 11 seropositive individuals were asymptomatic and 7 had mild symptoms. Thus, out of 100 participants enrolled in the second sampling, a total of 73 were previously tested positive for SARS-CoV-2 infection either using a RT-PCR or a serological test, or both. Sixty-seven of them were identified as seropositive 6 months after the outbreak, representing 92% (CI 83–96%) of tested SARS-CoV-2-positive individuals (n = 73). From a total of 67 seropositive participants, 43 (64%, CI 52–75%) were IgG-positive and 24 (36%, CI 25–48%) were positive for both IgG and IgM antibodies against SARS-CoV-2 (Table 3).

Thirty-nine individuals who were enrolled in the first serological testing on 17 June 2020 (2 months after the outbreak) participated in the second sampling on 20 October 2020 (6 months after the outbreak). Thirty-four out of 39 (87%, CI 73–95%) were found to be seropositive both at the first and the second serological testing. From this group, 13 had only IgG at both testing procedures. Twenty participants were found to be IgG/IgM-positive at the first sampling, whereas 13 of them were still IgG/IgM-positive and 7 were only IgG-positive at the second sampling. Importantly, all 34 participants of the study that were seropositive during the first test were also seropositive during the second test indicating the persistence of detectable antibody levels for at least 6 months (100%, CI 88–100%) (Table 3).

In the period of May 2020 to May 2021, all employees of the sewing company (n = 300) were periodically tested using RT-PCR for SARS-CoV-2 infection. No infection cases were reported between May 2020 and October 2020. Therefore, seropositivity due to new infection cases in the study cohort is unlikely.

### 3.5. Seropositivity Pattern 13 Months after the Outbreak

The third procedure of serological testing was performed 13 months after the outbreak and included 97 participants (median age—47 years, female—83.5%) who had been tested for SARS-CoV-2 infection using RT-PCR in April 2020 and then periodically (every 2 weeks) tested via PCR until May 2021. Blood specimens were tested both using a rapid serological test and a quantitative ELISA. From 97 participants of the third serological testing, 63 had a previous positive RT-PCR test in April 2020 (Figure 2a). At the time of third testing (12 May 2021), 16 out of 63 participants had received 1 or 2 doses of Comirnaty vaccine, and 47 were not vaccinated (Table 4). As expected, all previously infected and vaccinated individuals (n = 16) had virus-specific antibodies both using the rapid test and the ELISA.

**Figure 3:**
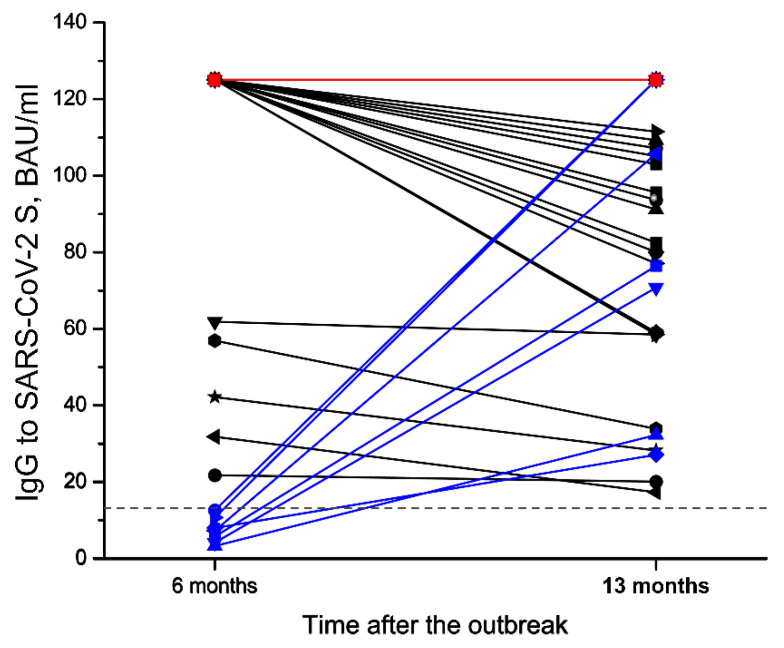
The levels of SARS-CoV-2-specific IgG in a group of previously infected non-vaccinated participants (n = 39) including 1 reinfection case (red line) in the second and the third serological testing. Between the 6th and 13th month after SARS-CoV-2 infection, a decline of virus-specific IgG levels was observed in 17 individuals (black lines); the IgG levels remained stable in 14 individuals (overlapping red lines) and increased in 7 individuals (blue lines).

In a group of study participants who recovered from SARS-CoV-2 infection and remained non-vaccinated (n = 47), one reinfection case was diagnosed using RT-PCR on 24 December 2020. In April 2020, this participant recovered from a severe COVID-19 (pneumonia, hospitalization, oxygen supply) and had high levels of virus-specific antibodies when tested 2 months and 6 months after the outbreak. The reinfection was asymptomatic and the IgG levels determined on 12 May 2021 were similar to those determined on 20 October 2020 (Figure 3, red line). Excluding the known reinfection case, 38 out of 46 in the group of previously infected non-vaccinated participants were seropositive (83%, CI 69–91%) (Table 4). All seropositive participants of this group were also documented as seropositive either in the first, second or both previous tests, which indicates the persistence of virus-specific antibodies for at least 13 months in the majority of the cases.

In the aforementioned group of recovered individuals, both asymptomatic and symptomatic SARS-CoV-2 infections of different severities were reported during the first outbreak (April 2020) (Table 4). In 31 out of 38 participants who had antibodies 13 months after the outbreak, the levels of SARS-CoV-2-specific IgG remained stable or decreased between 20 October 2020 and 12 May 2021, while 7 participants had higher IgG levels as compared to the second test (Figure 3, blue lines), suggesting a possible exposure to the virus during the second outbreak in the company in December 2020 (see Figure 1). None of them reported experiencing any symptoms or had a positive COVID-19 test.

Thirty-four out of 97 participants enrolled in the third testing procedure were negative for SARS-CoV-2 using RT-PCR during the outbreak in April 2020 and were also seronegative during the first and the second serological tests (Table 4). Seventeen out of 34 participants of this group were diagnosed with either asymptomatic or symptomatic SARS-CoV-2 infection via the periodic RT-PCR testing during the second wave of the pandemic, from November to December 2020 (Figure 2b).

Seven participants out of 34 were vaccinated with Comirnaty vaccine by the time of the third testing. As expected, all of them were found to be seropositive. Ten participants out of 34 had a negative RT-PCR result during periodic testing and had not been vaccinated (Table 4, Figure 2b). However, only 6 of them were seronegative at the time of the third testing and 4 were found to be seropositive without any previous symptoms, which may indicate an overlooked asymptomatic SARS-CoV-2 infection during the second wave of the pandemic characterized by high numbers of infection cases.

## 4. Discussion

Data on the persistence of the SARS-CoV-2-specific immune response are of great importance for managing the pandemic and planning further measures for increasing the population’s resistance to the virus. To date, both sero-epidemiological and targeted cohort studies from different countries have reported detectable levels of virus-specific antibodies and duration of the protective immune response from 3 to 13 months after infection [6,7,10,12,13,17,19,21,24,25]. Previous cohort studies on the formation and persistence of SARS-CoV-2-specific humoral immune response mainly included healthcare workers as a target group having the highest risk of being exposed to SARS-CoV-2 [13,14,16,19,20,24,26,27]. However, a long-term monitoring of antibody levels in naturally infected healthcare workers seems to be difficult or even impossible after the start of a global vaccination, as the healthcare sector is considered a priority group for vaccination in many countries [28]. Therefore, there are limited data on the persistence of SARS-CoV-2-specific antibodies over a prolonged period after infection.

In the current study, we investigated the seropositivity pattern at different intervals (2, 6 and 13 months) after a well-documented outbreak in a private Lithuanian company where about one-third of the employees (94 out of 300) had been diagnosed SARS-CoV-2-positive using RT-PCR during the first wave of the pandemic (April 2020). No additional cases were reported in between the first and second serological tests before the biggest COVID-19 wave in the country was documented in November–December 2020, allowing us to study different aspects of antibody persistence.

At the date of the first serological test conducted 2 months after the outbreak (17 June 2020), there were limited data on the seroconversion rate after asymptomatic or very mild SARS-CoV-2 infection. To address this question, we compared the pattern of seropositivity in participants who had recovered from SARS-CoV-2 infection of different severity—from asymptomatic infection to severe COVID-19 requiring hospitalization. From 50 individuals who agreed to participate in the first testing, 45 had a positive RT-PCR result during the outbreak and 34 of them self-reported either asymptomatic or very mild or mild infection, while 11 self-reported moderate to severe COVID-19. The seropositivity rate after an asymptomatic SARS-CoV-2 infection was lower when compared to symptomatic infection of different severity, which is in line with other studies [26]. Another issue addressed during the first serological testing was related to the persistence of virus-specific IgM and its potential diagnostic value for an acute SARS-CoV-2 infection. More than one half of seropositive individuals (22 out of 41) had both IgG and IgM virus-specific antibodies indicating the persistence of IgM over 2 months after infection. This confirms that virus-specific IgM is a non-appropriate serological marker for early SARS-CoV-2 infection [29].

The second serological testing included more participants (n = 100, among them 59 with a previous positive RT-PCR test and 41 with a previous negative RT-PCR test) and provided a comprehensive picture on the seropositivity rates in the following 6 months after the outbreak. The majority of previously infected participants (56 out of 59) had SARS-CoV-2-specific antibodies persisting for 6 months irrespective of the severity of infection (Figure 2a). All participants who were identified as seropositive 2 months after the outbreak (n = 34) maintained the seropositivity for 6 months after the confirmed SARS-CoV-2 infection. This result is in agreement with many other studies demonstrating the persistence of SARS-CoV-2-specific antibodies for at least 6 months after infection [9,12,13,15,16,21,27]. More than one third of seropositive individuals had virus-specific IgM along with IgG, which indicates that IgM may persist for 6 months, thus providing additional confirmation that IgM is not a suitable marker for identifying early infection due to expected high false-positivity for previously infected individuals [9,11,18]. Our findings on the persistence of virus-specific IgM are in line with previous studies that reported decreasing levels of anti-RBD IgM within 3–4 months post infection [30,31,32]. Moreover, the persistence of IgM in some individuals might be explained by the persistence of SARS-CoV-2 or its antigens as demonstrated in previous studies that detected viral antigen in biopsies from the gastrointestinal tract at 2.8–5.7 months after initial COVID-19 diagnosis [32].

In the second testing procedure, we identified 11 seropositive individuals who had had previous negative results with a RT-PCR test (Figure 2b). Although these participants were considered SARS-CoV-2-negative based on RT-PCR results, 7 of them experienced mild symptoms typical for SARS-CoV-2 infection and self-reported high-risk contacts during the outbreak. This suggests an overlooked previous infection despite periodic testing via RT-PCR during and after the outbreak. Unfortunately, serological testing was applied to a limited extent during the outbreak as only 40 workers out of 300 had been tested for virus-specific antibodies along with RT-PCR tests. Three of them were found to be seropositive but RT-PCR-negative in the first days of the outbreak, which may indicate them as a probable primary source of infection. In total, 14 cases of SARS-CoV-2 infection among RT-PCR-negative individuals were identified via serological testing either during the outbreak (n = 3) or 2 months after the outbreak in our study (n = 11). These findings indicate that serological testing is highly useful to confirm previous SARS-CoV-2 infection that has not been identified by RT-PCR tests. Moreover, serological testing in outbreak zones may reveal the primary source of infection and indicate the directions of virus spread when the virus is no longer detectable via RT-PCR, thus providing valuable information for epidemiological assessment.

The third serological testing (n = 97) more than one year after the outbreak allowed the durability of the humoral immune response and its dynamics to be evaluated during the second wave of the pandemic characterized by high numbers of active infection cases, especially in November‒December 2020 (see Figure 1). Before the third testing, COVID-19 vaccine was already available and one quarter (16 out of 63) of previously infected participants enrolled in the third testing were vaccinated by that time (Figure 2a). Thus, the third serological testing included 47 non-vaccinated participants with a confirmed SARS-CoV-2 infection during the outbreak in April 2020. In this group, 1 case of an asymptomatic reinfection was reported on 24 December 2020, despite the presence of virus-specific IgG on 24 October 2020. Excluding the known reinfection case, the seropositivity rate after 13 months was high with antibodies present in 38 out of 46 participants suggesting that detectable levels of virus-specific antibodies persist in a majority of the cases. The comparison of ELISA results 6 months and 13 months after the outbreak revealed sustained IgG levels in 14 participants and a decline of IgG levels in 17 participants, which is in agreement with other studies indicating a gradual decline of circulating antibodies after infection [12,16,17,24,33,34]. Interestingly, the levels of virus-specific IgG increased in 7 participants without any documented reinfection using periodically performed RT-PCR tests or self-reported symptoms. An increase in virus-specific IgG levels may indicate an asymptomatic exposure to SARS-CoV-2 between the second and the third serological testing procedures, which was highly likely during the second wave of the pandemic. An increased risk of SARS-CoV-2 infection within the period of November–December 2020 was confirmed by 17 new infection cases among 34 participants of our study that had had a negative RT-PCR result in April 2020 and were found to be seronegative in previous serological testing procedures (Figure 2b). As compared to only one reinfection case in a group of 47 seropositive participants, the high rate of new infection cases in seronegative participants supports data on the sustainable protective immune response after SARS-CoV-2 infection [12,14,35]. Further studies on such a “boosting” effect where asymptomatic SARS-CoV-2 infection of either previously infected or vaccinated individuals contributes to a protective immunity is very important as it has ramifications for longer term vaccination strategy.

Due to the company profile (sewing industry), the majority of the participants of our study (>80%) were female, which might be a potential source of bias when analysing the dynamics of antibody response post infection. Previous studies suggest that women mount stronger immune responses to infections and vaccinations, which is explained by sex-based differences in genes, sex hormones, and the microbiome underlying the host immune response [36]. In line with these observations, some studies demonstrated a more robust antibody response in females and a faster decline in virus-specific IgG levels in males after SARS-CoV-2 infection [37,38]. However, other studies did not find a statistically significant differences in the amount of SARS-CoV-2-specific IgG or IgM between male and female patients [39,40].

Summarizing, our study revealed the persistence of SARS-CoV-2-specific antibodies within a prolonged period of time (2 to 13 months) after an outbreak in a private sewing company in Lithuania during the first wave of pandemic. The strengths of our study are a long monitoring period and a well-documented cohort with the first infection cases properly described and followed by a periodic RT-PCR testing allowing new infection cases to be identified. Moreover, a relatively high number of study participants (n = 47) remained non-vaccinated despite a global vaccination program in the country and a wide accessibility of COVID-19 vaccines thus allowing the risk of reinfection and changes in antibody levels during the subsequent waves of the pandemic to be evaluated. In addition, a combination of a rapid IgM/IgG test targeting SARS-CoV-2 S1 and N proteins and a quantitative ELISA for S-specific IgG provided data on the persistence of both IgM and IgG and confirmed the previously reported observations on the durability of IgM response. Our study is different from other studies that mainly conducted serological monitoring in the healthcare sector, nursing homes or household settings representing high-risk zones for SARS-CoV-2 infection [41].

This study has some limitations. First, we did not test the neutralizing activities of virus-specific antibodies and did not investigate the dynamics of immune memory cells, therefore, their contributions to protective immunity are unknown. Second, due to the small sample size and a predominance of female participants in the study cohort, it is difficult to conclude whether the differences in new infection cases among seronegative (17 cases out of 34) and seropositive (1 case out of 47) individuals were determined by the sustainable antibody response or by other factors.

## Figures and Tables

**Table 1:** The characteristics of the study cohort.

Testing Date	Sample Size	Age of Study Participants					% of Female Participants
		Mean Age	SD	Median Age	IQR	Range (Years)	
17 June 2020	50	45.0	10.8	46	40–53	18–65	89.66
20 October 2020	100	45.1	10.8	46	40–53	18–65	88.89
12 May 2021	97	46.1	10.8	47	41–54	18–66	83.50

**Table 2:** Summarized results of the first serologic testing 2 months after the outbreak (17 June 2020).

	The Results of SARS-CoV-2-Specific RT-PCR Test at the Time of the Outbreak (April 2020)					
	Negative	Positive				
Classification of Symptoms	None	None	Very Mild	Mild	Moderate	Severe
Group Size	n = 5	n = 12	n = 13	n = 9	n = 8	n = 3
Only IgG+	0	4	8	3	3	1
Only IgM+	0	0	0	0	0	0
IgG+/IgM+	0	5	5	5	5	2
IgG−/IgM−	5	3	0	1	0	0
The number of seropositive individuals	0	9	13	8	8	3
Seropositivity rate, %	0	75	100	89	100	100

**Table 3:** Summarized results of the second serologic testing 6 months after the outbreak (20 October 2020).

**Total number of participants of the second serologic testing**	**100**
The number of participants with a previous * positive RT-PCR result	59
The number of participants with a previous * negative RT-PCR result	41
Among them, identified as seropositive	11
Among them, identified as seronegative	30
The number of seropositive participants	67
The number of seronegative participants	33
Seropositive with a previous * positive RT-PCR result	56
Self-reported asymptomatic infection	11
Self-reported mild or very mild symptoms	33
Self-reported moderate or severe symptoms	12
Seropositive with a previous * negative RT-PCR result	11
Both IgG+/IgM+	24
Only IgM+	0
Only IgG+	43
**The number of participants enrolled both into the first (2 months after the outbreak) and the second (6 months after the outbreak) serologic testing**	**39**
Seronegative both in the first and the second serologic testing	5
Seropositive in the second serologic testing	34
Seropositive in the first serologic testing	34
Only IgM+ in the first serologic testing	0
Only IgG+ in the first serologic testing	13
From them, only IgG+ in the second serologic testing	13
Both IgG+/IgM+ in the first serologic testing	21
From them, both IgG+/IgM+ in the second serologic testing	14
From them, only IgG+ positive in the second serologic testing	7

* SARS-CoV-2-specific RT-PCR test performed during the outbreak (April 2020).

**Table 4:** Summarized results of the third serologic testing 13 months after the outbreak (12 May 2021).

Total Number of Participants of the Third Serologic Testing	97
The number of participants with a previous * negative RT-PCR result	34
No confirmed SARS-CoV-2 infection and vaccination record	10
Confirmed SARS-CoV-2 infection in November‒December 2020	17
Vaccinated in April–May 2021	7
The number of participants with a previous * positive RT-PCR result	63
Vaccinated in April–May 2021	16
Non-vaccinated by the date of the third serologic testing	47
Confirmed SARS-CoV-2 infection in December 2020	1
Seropositive	38
Seronegative	8

* SARS-CoV-2-specific RT-PCR test performed during the outbreak (April 2020).

## References

[1] Xiao A.T., Gao C., Zhang S. (2020). Profile of specific antibodies to SARS-CoV-2: The first report. J. Infect..

[2] Beaudoin-Bussières G., Laumaea A., Anand S.P., Prévost J., Gasser R., Goyette G., Medjahed H., Perreault J., Tremblay T., Lewin A. (2020). Decline of humoral responses against SARS-CoV-2 spike in convalescent individuals. MBio.

[3] Peeling R.W., Wedderburn C.J., Garcia P.J., Boeras D., Fongwen N., Nkengasong J., Sall A., Tanuri A., Heymann D.L. (2020). Serology testing in the COVID-19 pandemic response. Lancet Infect. Dis..

[4] Long Q.-X., Liu B.-Z., Deng H.-J., Wu G.-C., Deng K., Chen Y.-K., Liao P., Qiu J.-F., Lin Y., Cai X.-F. (2020). Antibody responses to SARS-CoV-2 in patients with COVID-19. Nat. Med..

[5] Iyer A.S., Jones F.K., Nodoushani A., Kelly M., Becker M., Slater D., Mills R., Teng E., Kamruzzaman M., Garcia-Beltran W.F. (2020). Persistence and decay of human antibody responses to the receptor binding domain of SARS-CoV-2 spike protein in COVID-19 patients. Sci. Immunol..

[6] Röltgen K., Powell A.E., Wirz O.F., Stevens B.A., Hogan C.A., Najeeb J., Hunter M., Wang H., Sahoo M.K., Huang C.H. (2020). Defining the features and duration of antibody responses to SARS-CoV-2 infection associated with disease severity and outcome. Sci. Immunol..

[7] Crawford K.H.D., Dingens A.S., Eguia R., Wolf C.R., Wilcox N., Logue J.K., Shuey K., Casto A.M., Fiala B., Wrenn S. (2021). Dynamics of Neutralizing Antibody Titers in the Months After Severe Acute Respiratory Syndrome Coronavirus 2 Infection. J. Infect. Dis..

[8] Isho B., Abe K.T., Zuo M., Jamal A.J., Rathod B., Wang J.H., Li Z., Chao G., Rojas O.L., Bang Y.M. (2020). Persistence of serum and saliva antibody responses to SARS-CoV-2 spike antigens in COVID-19 patients. Sci. Immunol..

[9] Wang H., Yuan Y., Xiao M., Chen L., Zhao Y., Zhang H., Long P., Zhou Y., Xu X., Lei Y. (2021). Dynamics of the SARS-CoV-2 antibody response up to 10 months after infection. Cell. Mol. Immunol..

[10] Wang K., Long Q.-X., Deng H.-J., Hu J., Gao Q.-Z., Zhang G.-J., He C.-L., Huang L.-Y., Hu J.-L., Chen J. (2020). Longitudinal Dynamics of the Neutralizing Antibody Response to Severe Acute Respiratory Syndrome Coronavirus 2 (SARS-CoV-2) Infection. Clin. Infect. Dis..

[11] Liu C., Yu X., Gao C., Zhang L., Zhai H., Hu Y., Liu E., Wang Q., Gao Y., Wei D. (2021). Characterization of antibody responses to SARS-CoV-2 in convalescent COVID-19 patients. J. Med. Virol..

[12] Feng C., Shi J., Fan Q., Wang Y., Huang H., Chen F., Tang G., Li Y., Li P., Li J. (2021). Protective humoral and cellular immune responses to SARS-CoV-2 persist up to 1 year after recovery. Nat. Commun..

[13] Gallais F., Gantner P., Bruel T., Velay A., Planas D., Wendling M.-J., Bayer S., Solis M., Laugel E., Reix N. (2021). Evolution of antibody responses up to 13 months after SARS-CoV-2 infection and risk of reinfection. EBioMedicine.

[14] Lumley S.F., Wei J., O’Donnell D., Stoesser N.E., Matthews P.C., Howarth A., Hatch S.B., Marsden B.D., Cox S., James T. (2021). The Duration, Dynamics, and Determinants of Severe Acute Respiratory Syndrome Coronavirus 2 (SARS-CoV-2) Antibody Responses in Individual Healthcare Workers. Clin. Infect. Dis..

[15] Petersen M.S., Hansen C.B., Kristiansen M.F., Fjallsbak J.P., Larsen S., Hansen J.L., Jarlhelt I., Pérez-Alós L., á Steig B., Christiansen D.H. (2021). SARS-CoV-2 Natural Antibody Response Persists for at Least 12 Months in a Nationwide Study From the Faroe Islands. Open Forum Infect. Dis..

[16] Varona J.F., Madurga R., Peñalver F., Abarca E., Almirall C., Cruz M., Ramos E., Castellano-Vazquez J.M. (2021). Kinetics of anti-SARS-CoV-2 antibodies over time. Results of 10 month follow up in over 300 seropositive Health Care Workers. Eur. J. Intern. Med..

[17] Dan J.M., Mateus J., Kato Y., Hastie K.M., Yu E.D., Faliti C.E., Grifoni A., Ramirez S.I., Haupt S., Frazier A. (2021). Immunological memory to SARS-CoV-2 assessed for up to 8 months after infection. Science.

[18] Anand S.P., Prévost J., Nayrac M., Beaudoin-Bussières G., Benlarbi M., Gasser R., Brassard N., Laumaea A., Gong S.Y., Bourassa C. (2021). Longitudinal analysis of humoral immunity against SARS-CoV-2 Spike in convalescent individuals up to 8 months post-symptom onset. Cell Rep. Med..

[19] Duysburgh E., Mortgat L., Barbezange C., Dierick K., Fischer N., Heyndrickx L., Hutse V., Thomas I., Van Gucht S., Vuylsteke B. (2021). Persistence of IgG response to SARS-CoV-2. Lancet Infect. Dis..

[20] Choudhry N., Drysdale K., Usai C., Leighton D., Sonagara V., Buchanan R., Nijjar M., Thomas S., Hopkins M., Cutino-Moguel T. (2021). Disparities of SARS-CoV-2 Nucleoprotein-Specific IgG in Healthcare Workers in East London, UK. Front. Med..

[21] Wisnivesky J.P., Stone K., Bagiella E., Doernberg M., Mendu D.R., Lin J.J., Kale M. (2021). Long-term Persistence of Neutralizing Antibodies to SARS-CoV-2 Following Infection. J. Gen. Intern. Med..

[22] Stringhini S., Zaballa M.-E., Pullen N., de Mestral C., Perez-Saez J., Dumont R., Picazio A., Pennacchio F., Dibner Y., Yerly S. (2021). Large variation in anti-SARS-CoV-2 antibody prevalence among essential workers in Geneva, Switzerland. Nat. Commun..

[23] Mattiuzzo G., Bentley E.M., Hassall M., Routley S., Richardson S., Bernasconi V., Kristiansen P., Harvala H., Roberts D., Semple M.G. Establishment of the WHO International Standard and Reference Panel for anti-SARS-CoV-2 antibody on behalf of the ISARIC4C Investigators. Proceedings of the Expert Committee On Biological Standardization.

[24] Seow J., Graham C., Merrick B., Acors S., Pickering S., Steel K.J.A., Hemmings O., O’Byrne A., Kouphou N., Galao R.P. (2020). Longitudinal observation and decline of neutralizing antibody responses in the three months following SARS-CoV-2 infection in humans. Nat. Microbiol..

[25] Wajnberg A., Amanat F., Firpo A., Altman D.R., Bailey M.J., Mansour M., McMahon M., Meade P., Mendu D.R., Muellers K. (2020). Robust neutralizing antibodies to SARS-CoV-2 infection persist for months. Science.

[26] Horton D.B., Barrett E.S., Roy J., Gennaro M.L., Andrews T., Greenberg P., Bruiners N., Datta P., Ukey R., Velusamy S.K. (2021). Determinants and Dynamics of SARS-CoV-2 Infection in a Diverse Population: 6-Month Evaluation of a Prospective Cohort Study. J. Infect. Dis..

[27] Robertson L.J., Moore J.S., Blighe K., Ng K.Y., Quinn N., Jennings F., Warnock G., Sharpe P., Clarke M., Maguire K. (2021). Evaluation of the IgG antibody response to SARS-CoV-2 infection and performance of a lateral flow immunoassay: Cross-sectional and longitudinal analysis over 11 months. BMJ Open.

[28] Sarantopoulos A., Brown D., Wiedermann U., Dominguez C.A., Bogdan C., Gürsel İ., Janković S., LeClerc C., Locati M., Spurkland A. (2021). The EFIS vaccination task force expert report. Eur. J. Immunol..

[29] The Use of Antibody Tests for SARS-COV-2 in the Context of Digital Green Certificates. https://www.ecdc.europa.eu/en/publications-data/use-antibody-tests-sars-cov-2-context-digital-green-certificates.

[30] Jiang X.L., Wang G.L., Zhao X.N., Yan F.H., Yao L., Kou Z.Q., Ji S.X., Zhang X.L., Li C.B., Duan L.J. (2021). Lasting antibody and T cell responses to SARS-CoV-2 in COVID-19 patients three months after infection. Nat. Commun..

[31] Secchi M., Bazzigaluppi E., Brigatti C., Marzinotto I., Tresoldi C., Rovere-Querini P., Poli A., Castagna A., Scarlatti G., Zangrillo A. (2020). COVID-19 survival associates with the immunoglobulin response to the SARS-CoV-2 spike receptor binding domain. J. Clin. Investig..

[32] Gaebler C., Wang Z., Lorenzi J.C.C., Muecksch F., Finkin S., Tokuyama M., Cho A., Jankovic M., Schaefer-Babajew D., Oliveira T.Y. (2021). Evolution of antibody immunity to SARS-CoV-2. Nature.

[33] Ibarrondo F.J., Fulcher J.A., Goodman-Meza D., Elliott J., Hofmann C., Hausner M.A., Ferbas K.G., Tobin N.H., Aldrovandi G.M., Yang O.O. (2020). Rapid Decay of Anti–SARS-CoV-2 Antibodies in Persons with Mild COVID-19. Engl. J. Med..

[34] Ward H., Cooke G., Atchison C., Whitaker M., Elliott J., Moshe M., Brown J.C., Flower B., Daunt A., Ainslie K. (2020). Declining prevalence of antibody positivity to SARS-CoV-2: A community study of 365,000 adults. medRxiv.

[35] Khoury D.S., Cromer D., Reynaldi A., Schlub T.E., Wheatley A.K., Juno J.A., Subbarao K., Kent S.J., Triccas J.A., Davenport M.P. (2021). Neutralizing antibody levels are highly predictive of immune protection from symptomatic SARS-CoV-2 infection. Nat. Med..

[36] Gadi N., Wu S.C., Spihlman A.P., Moulton V.R. (2020). What’s Sex Got to Do With COVID-19? Gender-Based Differences in the Host Immune Response to Coronaviruses. Front. Immunol..

[37] Zeng F., Dai C., Cai P., Wang J., Xu L., Li J., Hu G., Wang Z., Zheng F., Wang L. (2020). A comparison study of SARS-CoV-2 IgG antibody between male and female COVID-19 patients: A possible reason underlying different outcome between sex. J. Med. Virol..

[38] Grzelak L., Velay A., Madec Y., Gallais F., Staropoli I., Schmidt-Mutter C., Wendling M.J., Meyer N., Planchais C., Rey D. (2021). Sex Differences in the Evolution of Neutralizing Antibodies to Severe Acute Respiratory Syndrome Coronavirus 2. J. Infect. Dis..

[39] Takahashi T., Ellingson M.K., Wong P., Israelow B., Lucas C., Klein J., Silva J., Mao T., Oh J.E., Tokuyama M. (2020). Sex differences in immune responses that underlie COVID-19 disease outcomes. Nature.

[40] Zeng F., Wu M., Wang J., Li J., Hu G., Wang L. (2021). Over 1-year duration and age difference of SARS-CoV-2 antibodies in convalescent COVID-19 patients. J. Med. Virol..

[41] Miller E., Waight P.A., Andrews N.J., McOwat K., Brown K.E., Höschler K., Ijaz S., Letley L., Haskins D., Sinnathamby M. (2021). Transmission of SARS-CoV-2 in the household setting: A prospective cohort study in children and adults in England. J. Infect..

